# Gene flow and population structure of a solitary top carnivore in a human-dominated landscape

**DOI:** 10.1002/ece3.1322

**Published:** 2014-12-24

**Authors:** Jeannine S McManus, Desiré L Dalton, Antoinette Kotzé, Bool Smuts, Amy Dickman, Jason P Marshal, Mark Keith

**Affiliations:** 1 School of Animal, Plant and Environmental SciencesUniversity of the Witwatersrand Private Bag X3, Johannesburg, 2050, South Africa; 2 Landmark FoundationP.O. Box 22, Riversdale, 6670, South Africa; 3 National Zoological Gardens of South AfricaP.O. Box 754, Pretoria, 0001, South Africa; 4 Genetics Department, University of the Free StateP.O. Box 339, Bloemfontein, 9300, South Africa; 5 WildCRU, Oxford UniversityAbingdon Road, Tubney, Abingdon, OX13 5QL, UK; 6 Centre for Wildlife Management, University of PretoriaPrivate Bag X20 Hatfield, Pretoria 0028, South Africa

**Keywords:** Gene flow, genetic differentiation, habitat fragmentation, *Panthera pardus*, population structure

## Abstract

While African leopard populations are considered to be continuous as demonstrated by their high genetic variation, the southernmost leopard population exists in the Eastern and Western Cape, South Africa, where anthropogenic activities may be affecting this population's structure. Little is known about the elusive, last free-roaming top predator in the region and this study is the first to report on leopard population structuring using nuclear DNA. By analyzing 14 microsatellite markers from 40 leopard tissue samples, we aimed to understand the populations' structure, genetic distance, and gene flow (*Nm*). Our results, based on spatially explicit analysis with Bayesian methods, indicate that leopards in the region exist in a fragmented population structure with lower than expected genetic diversity. Three population groups were identified, between which low to moderate levels of gene flow were observed (*Nm* 0.5 to 3.6). One subpopulation exhibited low genetic differentiation, suggesting a continuous population structure, while the remaining two appear to be less connected, with low emigration and immigration between these populations. Therefore, genetic barriers are present between the subpopulations, and while leopards in the study region may function as a metapopulation, anthropogenic activities threaten to decrease habitat and movement further. Our results indicate that the leopard population may become isolated within a few generations and suggest that management actions should aim to increase habitat connectivity and reduce human–carnivore conflict. Understanding genetic diversity and connectivity of populations has important conservation implications that can highlight management of priority populations to reverse the effects of human-caused extinctions.

## Introduction

Ensuring the maintenance of genetic diversity and connectivity among populations facilitates the continuation of dynamic evolutionary and ecological processes. Genetic data provide insights into the population structure of a species and the rate of genetic movement between populations, which helps to determine the possibility of local adaptation and of adaptive evolution in complex landscapes (Hanski and Gilpin 1991). Whether continuous or discrete, population structures are influenced by a variety of factors, including species-innate traits such as dispersal behavior (Wayne and Koepfli 1996; Sork et al. 1999), climatic factors (Stenseth et al. 2004), and geographic features that may facilitate or constrain movement (Ginsberg and Milner-Gulland 1994; Woodroffe and Ginsberg 1998; Frankham 2005). Metapopulations experience local extinctions and recolonizations of subpopulations through immigration and emigration (Groom et al. 2006). Therefore, high mobility and dispersal influence the long-term survival and adaptation of species and facilitate population persistence. Large carnivores often have the ability to traverse extensive distances and can occupy a variety of environmental conditions (Sweanor et al. 2000; Sunquist and Sunquist 2002). However, even where species are highly mobile, discontinuous habitat and anthropogenic-associated barriers, such as major roads, monoculture, and human-caused mortality, constrain movements and reduce population densities (Walker et al. 2000; Sinclair et al. 2001; Woodroffe et al. 2005; Loxterman 2011). The proportion of declines in carnivore populations caused by human-induced mortality has been compared with declines in actively culled populations (Wielgus and Bunnell 1994; Powell et al. 1996) and is considered, more than any other factor, to be the main cause of extinctions for small, isolated populations (Woodroffe and Ginsberg 1998).

If genetic transfer between populations is impeded, two major genetic threats may present themselves: firstly, alleles become randomly fixed or are lost due to genetic drift, and secondly, harmful mutations accumulate. Subpopulations may not recover from such impacts, degrading the persistence of metapopulations (Sweanor et al. 2000) and reducing the populations' ability to adapt to changing environmental conditions, diseases, and other stochastic events that threaten their survival (Keller and Waller 2002; Frankham 2005; O'Brien and Johnson 2005). Leopards are highly mobile and are considered to be the most adaptable felid in the world, able to occupy most environments except true desert (Sunquist and Sunquist 2002). In much of their range, leopards are the last remaining free-roaming top predator. This top predator status influences community structure in lower trophic levels, driving biodiversity (Carroll et al. 2001; Noss et al. 2002). Despite this important ecological role, the solitary and elusive nature of leopards has made research on the species difficult, resulting in potentially inappropriate management actions, such as lethal predator control (human–carnivore conflict; trophy hunting) when the population may already be vulnerable to extinction. Recently, however, the use of DNA has provided an opportunity to increase our knowledge of this species. Understanding the population structure and diversity within and between populations is crucial to estimate the extent of divergence among populations, recognize evolutionary significant units, preserve genetic diversity among remnant populations, and highlight management of priority populations to reverse the effects of human-caused extinctions (O'Brien and Johnson 2005).

Studies examining nuclear DNA from leopards in central Africa indicate that populations are continuous, with high levels of genetic heterozygosity (Miththapala et al. 1996; Spong et al. 2000; Uphyrkina et al. 2001). In South Africa, one study used mitochondrial DNA (mtDNA) from 29 individuals sampled across the country (Martins 2006). These maternal phylogeographic outputs indicated high levels of genetic diversity overall, but genetic separation between the Western Cape province and other areas of South Africa (Martins 2006). However, the use of mtDNA for asymmetrical dispersing mammals, including leopards, where males disperse in order to mate, while females are philopatric (Bailey 1993; Sunquist and Sunquist 2002), may not represent the true situation (Melnick and Hoelzer 1992; Zhang and Hewitt 2003). An additional problem is the presence of mitochondrial pseudogenes in the nuclear genome that weaken the effectiveness of using mtDNA in population genetic studies (Zhang and Hewitt 2003). Therefore, the genetic status and population structuring of leopards in the region is not fully understood and can be questioned. The use of microsatellites provides more insight than does mtDNA into the genetic structure, gene flow, heterozygosity, and general population connectivity for closely related populations (Teske et al. 2012).

The southernmost part of South Africa (Eastern and Western Cape provinces) is characterized by a matrix of land uses, highly fragmented natural habitat and human–carnivore conflict, all of which result in carnivore mortality. Such landscape characteristics have resulted in reduced gene flow in other carnivore species around the world (Sinclair et al. 2001; McRae et al. 2005). We investigate the population structure using spatially explicit methods to determine the genetic distance, gene flow, and heterozygosity of 40 leopards by analyzing 14 microsatellite markers. We present results from the Eastern and Western Cape, South Africa and consider the conservation implications these have for isolated carnivore populations in a human-dominated landscape.

## Materials and Methods

### Study area and sampling

**Figure 1:**
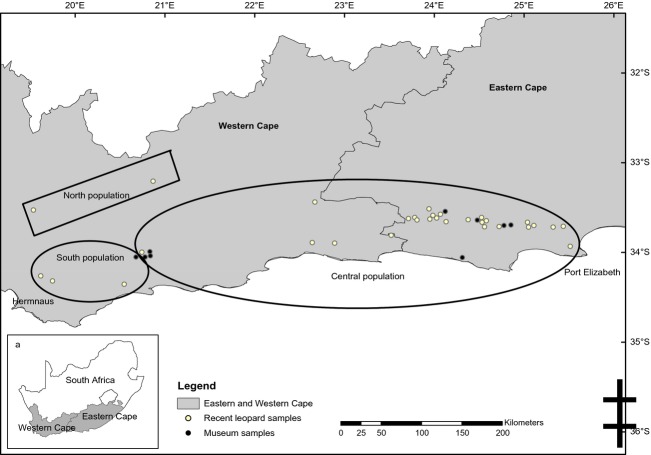
Study area within South Africa (a) where shading indicates the location of the Eastern and Western Cape. The location of 40 samples (recent and museum) collected from the study area. Sample locations enveloped by black polygons denote which of the three subpopulations (*K* = 3) the sample was assigned to as defined by GENELAND “north population,” “south population,” and “central population.”

Forty tissue samples were collected across the Eastern and Western Cape provinces (33°11′–33°23′S and 25°53′–18°53′E; Fig.1). Nine samples were from museums (oldest sample from 1976, most recent 1996), while 31 were collected from free-ranging leopards during capture and immobilization of leopards associated with a broader ecological study during 2007–2013 (Fig.1). All samples were accurately georeferenced. Leopards were captured in walk-in, two fall-door cages 2 m × 800 mm × 800 mm. Cages were set where leopard activity was present (spoor, scat, scrapings), but no baits or lures were used in cages in order to reduce bycatch. Immobilizations were undertaken by a qualified veterinarian, using a drug combination of Zoletol–Medetomidine at a standard dosage (1–2 mg/kg). Induction times averaged seven minutes, minimizing stress to the leopards. Recovery of animals from the reversal drugs averaged eight minutes. Samples were stored in a high-salt solution (Seutin et al. 1991) that retained good-quality DNA. No samples were collected from captive individuals.

### DNA extraction and amplification

Genomic DNA was extracted from tissue using the QIAGEN DNeasy® Blood and Tissue Kit (GmbH, Hilden, Germany), following the manufacturer's protocol. Leopard samples were analyzed using 14 microsatellite loci developed from the domestic cat (Menotti-Raymond and O'Brien 1995). Fourteen markers were optimized: FCA024, FCA032, FCA082, FCA085, FCA096, FCA129, FCA133, FCA161, FCA191, FCA211, FCA224, FCA261, FCA275, and FCA391. Promega GoTaq® Flexi DNA Polymerase (Promega Corporation, Madison, WI, USA) was used for amplification in 12.5-*μ*L reactions. The final reaction conditions were as follows: 1 × PCR buffer, 1 mmol/L MgCl_2_, 200 *μ*mol/L of each dNTP, 10 pmol of each primer (forward and reverse), 1 U *Taq* DNA polymerase, and 50 ng genomic DNA template. PCR was conducted in the BOECO TC-PRO Thermal Cycler. The amplification conditions were as follows: five minutes at 95°C, 30 cycles for 30 sec at 95°C, 30 sec at 50–60°C, and 30 sec at 72°C, followed by extension at 72°C for 40 min. The dye-labeled PCR products of the microsatellite primer sets were pooled and diluted together based on size range and fluorescent dye, so that 3 to 6 loci could be multiplexed, electrophoresed, and subsequently analyzed in an ABI3130 automated sequencer. Microsatellite allele sizes were estimated by comparison with a LIZ™ 500 (ABI, Foster City, CA) internal size standard. Data were collected and analyzed using the ABI programs GENESCAN (version 1.2.2-1) and GENOTYPER (version 1.1). We conducted a minimum of two replicate PCRs per tissue sample per locus. Alleles included in the final consensus genotypes were observed twice; if observed once, additional replicates were conducted. We also included a negative and positive control in each PCR as checks for contamination and to ensure standardized genotypes among experiments.

### Molecular analysis

#### Population genetic analysis

MICRO-CHECKER 2.2.3 (Van Oosterhout et al. 2004) was used to detect possible genotyping errors, allele dropout, and nonamplified alleles (null alleles). This software package can estimate the frequency of null alleles and adjust the dataset to correct for genotyping errors. Deviations from Hardy–Weinberg equilibrium (HWE) proportions were calculated using GenAlEx 6.5 (Peakall and Smouse 2006). Linkage disequilibrium between pairs of microsatellite loci was evaluated using Genepop 4.0 (Raymond and Rousset 1995). Associated probability values were corrected for multiple comparisons using Bonferroni adjustment for a significance level of 0.05. Levels of genetic variation were inferred from the average number of alleles per locus (A), the observed heterozygosity (H_O_), Nei's (1978) unbiased expected heterozygosity (H_E_), and percentage/number of private alleles, all of which were calculated using the software GenAlEx 6.5 (Peakall and Smouse 2006).

#### Global genetic structure of populations

Divergence of species can occur within a short time (Anderson et al. 2010), so determining whether population substructure resulted on an evolutionary scale or recently was important to estimate. We used a Bayesian clustering method implemented in the software program GENELAND, version 3.1.4 (Guillot et al. 2005), to determine the genetic structure of leopard. This program was able to identify divergence between populations as recently as four generations and found to be the best method available to determine contemporary population subdivision (e.g., Blair et al. 2012). Unlike STRUCTURE (Pritchard et al. 2000), GENELAND uses spatial location and genotypic data for all individuals to infer the number of population subdivisions and to assign individuals to each. In this way, we were able to identify cryptic patterns of structure where barriers in the fragmented landscapes may not have been obvious. *K* was determined across 10 iterations using GENELAND. All runs were conducted using 1,000,000 Markov chain Monte Carlo (MCMC) iterations. Genetic differentiation was examined between the inferred clusters using *F*-statistics calculated in GENEPOP (Raymond and Rousset 1995). Pairwise estimates of *F*_ST_ (Weir and Cockerham 1984) were calculated in order to determine genetic distance between clusters. The rate of gene flow across the sampled units was expressed as the number of migrants per generation, *Nm*, where *N* is the effective population size and *m* is the proportion of migration per generation. *Nm* is approximated by (1/*F*_ST_−1)/4 (Wright 1984; Slatkin 1987).

## Results

### Population genetic analysis

All 14 microsatellite loci were polymorphic. Mean *H*_o_ values ranged from very low to very high (0.35–0.88), *H*_e_ values ranged from 0.50 to 0.81, and the average number of alleles per locus was 6.5 (Table1). *H*_o_ values were lower than *H*_e_ in 10 of the markers, with an average observed heterozygosity level of 0.657. Pooling of the samples into one large population resulted in deviations from HWE for seven markers (Table1). In addition, null alleles were observed for one marker (FCA211) and linkage disequilibrium between markers was not observed. There are many possible explanations for departures from Hardy–Weinberg proportions, including natural selection, population subdivision, and null alleles. The high proportion of null alleles could be due to the markers used being developed for other species, which could result in high allele amplification failure due to mutations in primer locations. Previous studies on noninvasive samples collected from leopards that were amplified using cross-species markers identified an allelic dropout rate of 0–9% (Mondol et al. 2009). However, this estimate is probably higher than the actual rate. Thus, assuming that genotype errors were randomly distributed with respect to the population, this error rate is unlikely to bias our estimates of genetic diversity and divergence. The heterozygote deficiency was interpreted as the Wahlund effect (Wahlund, 1928), indicating the differentiation between leopard populations which were therefore analyzed separately.

**Table 1:** Genetic diversity measure across all leopard populations in South Africa. Null allele frequencies estimated with MICRO-CHECKER for 14 microsatellite loci. Hardy–Weinberg equilibrium values as calculated by GENEPOP

Locus	No. of alleles	*H*_o_	*H*_e_	Null allele frequency	HWE	*F*_IS_
FCA391	7	0.576	0.661	0.076	0.029*	0.129
FCA024	6	0.647	0.740	0.0606	0.772^NS^	0.125
FCA129	8	0.722	0.806	0.0467	0.002**	0.104
FCA032	6	0.641	0.766	0.075	0.000***	0.163
FCA082	7	0.730	0.759	0.0117	0.077^NS^	0.038
FCA275	4	0.350	0.499	0.1384	0.446^NS^	0.299
FCA191	7	0.650	0.641	−0.0363	0.677^NS^	−0.014
FCA133	6	0.611	0.698	0.0553	0.098^NS^	0.125
FCA161	8	0.625	0.617	−0.0029	0.000***	−0.013
FCA224	8	0.629	0.744	0.0582	0.000***	0.156
FCA085	7	0.882	0.768	−0.0823	0.412^NS^	−0.149
FCA211	6	0.577	0.793	0.131	0.001***	0.272
FCA261	6	0.750	0.799	0.0251	0.010*	0.061
FCA097	5	0.813	0.742	−0.0508	0.462^NS^	−0.095
Average	6.5	0.657	0.717	–	–	0.086

NS. Nonsignificant;

* *P* < 0.05;

** *P *≤ 0.01;

*** *P *≤ 0.001.

*H*_e_ values calculated to correct for uneven sample size. *F*_IS_ is the inbreeding coefficient.

### Genetic structure of populations

**Figure 2:**
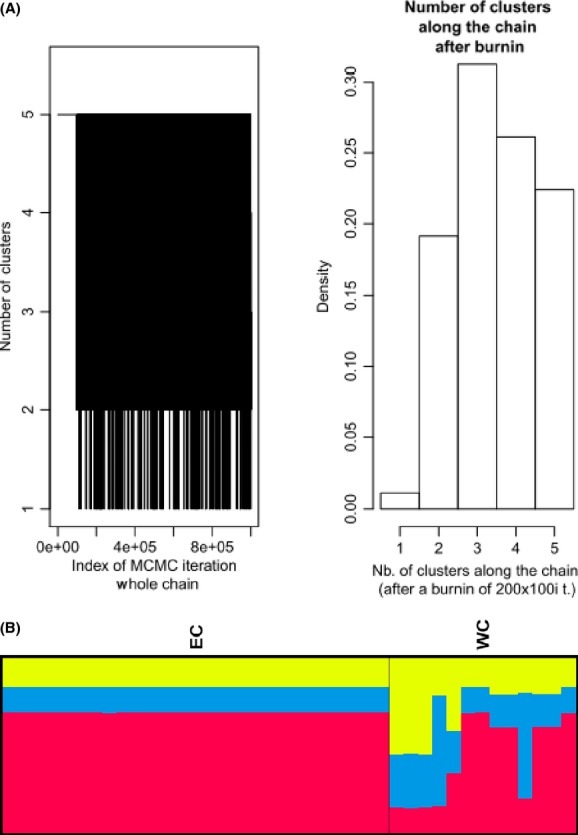
Estimated number of populations from GENELAND. (A) Posterior density distribution of the number of clusters estimated from GENELAND analysis. (B) Estimated population structure from GENELAND analyses for the model solutions *K* = 3. Each individual is represented by a thin horizontal line divided into *K* colored segments that represent the individual's estimated membership fractions in *K* clusters. Black lines separate individuals from different geographic areas labeled at the bottom.

This study provided important insights into the population structure and gene flow of leopards in the Eastern and Western Cape provinces of South Africa. All GENELAND runs produced a *K* = 3 population estimate (Fig.2). Based on the boundaries identified by GENELAND, clusters were labeled as follows: “south population,” “north population,” and “central population” (Fig.1). Supporting evidence was deducted from the AMOVA, as 12% of the variation was shared among the different localities. The high genetic distance measure between the central and north (*F*_ST_ = 0.30463; *P* = 0.000) and south and north (*F*_ST_ = 0.32943; *P* = 0.063) populations supports a strong substructuring of the diversity. Gene flow among these populations was therefore low (*Nm* 0.57 and 0.51, respectively). However, a moderate genetic distance measure was observed between the central and south populations (*F*_ST_ = 0.06360; *P* = 0.072), suggesting some gene flow between these populations (*Nm* 3.68). This may be explained by one museum sample, which formed part of the south population, collected in 1986 on the western edge of the large central population. This individual probably migrated between these two populations. Private alleles were observed in all three populations, namely 27 in the central population, three in the south population, and two in the north population.

## Discussion

Although leopards in Africa are considered to have high genetic diversity and little population structuring (Miththapala et al. 1996; Spong et al. 2000; Uphyrkina et al. 2001), significant population genetic structure of leopards has been detected in the southernmost part of Africa. Three subpopulations were identified in the region with moderate to low levels of genetic differentiation. These results indicate that leopards in the Eastern and Western Cape are not panmictic, despite their high mobility and environmental plasticity. Human disturbance, leading to contemporary landscape changes, may be responsible for this low gene flow.

Predictive habitat modeling for leopard indicates that topographic features such as mountain ranges, tall vegetation cover, and the close proximity of rivers promote leopard movement, while features fragmenting habitat, such as human-associated land uses and roads, are avoided (Gavashelishvili and Lukarevskiy 2008; Swanepoel et al. 2013). Urban areas and road networks have expanded, and agriculture has been intensified in the region in recent history, which has resulted in reduced gene flow and lowered genetic diversity for many species, including large mammals with longer life spans (Epps et al. 2005; Pilot et al. 2006; Holderegger and Di Giulio 2010). Reduced levels of genetic transfer can increase genetic differentiation within a few generations (Epps et al. 2005; Balkenhol et al. 2009), so the use of appropriate analytical tools to identify these recent divergences are important (Anderson et al. 2010; Blair et al. 2012). Leopards live for approximately eight to 12 years in the wild, and we could detect contemporary population divergences within the last 20 generations using Bayesian statistics and GENELAND (Blair et al. 2012).

Our findings highlight the sensitivity of leopard to landscape changes involving human occupation. The higher genetic differentiation observed in the north population (*F*_ST_ = 0.304–0.329) indicates that more habitat fragmentation exists between the north and the remaining populations. While the south and central populations indicate some gene flow between them (*Nm* 3.68), one sample forming part of the south population was collected in 1986 on the western edge of the central population. Therefore, the finding of lower genetic distance between the central and south populations may be due to remnant connectivity. The high number of private alleles maintained by the central population (*n* = 27), compared to both south and north populations, further suggests that the latter populations have been isolated from genetic transfer of the continuous central population and have undergone a severe genetic bottleneck effect.

These results indicate that the leopard population can broadly be described as a metapopulation (see Hanski and Gilpin 1991) with subpopulations in habitat patches separated by human-dominated landscape, linked to some degree by genetic flow. However, low gene flow and few private alleles indicate that the south and north populations may be functioning as sink populations, while the central population may have been a source in the recent past. Furthermore, the low genetic transfer between populations may reduce potential recolonization of extinct populations, posing direct threat to the observed populations (Levins 1970). Other fundamental characteristics of metapopulations have not been demonstrated, including independent dynamics among patches, natural extirpations, and natural recolonizations of extirpated populations (Harrison and Taylor 1997). Therefore, we suggest that the leopard population was previously a single population with patchy, or extinction-resistant distribution (Harrison 1991). As anthropogenic land use and human–carnivore conflict increased, this single large population became increasingly fragmented, resulting in semi-isolated to isolated populations surrounded by non-leopard habitat, as has happened to carnivores elsewhere (Beier 1995; Cegelski et al. 2003; McRae et al. 2005). As a result, leopard populations in the region may not function to the full extent as metapopulations. If isolation trends continue and gene flow further decreases between populations, they will become isolated and separate into distinct evolutionary units and possibly experience higher probabilities of extinction (Schaffer 1988).

Our findings are further supported by the lower than expected heterozygosity present in 10 of the 14 markers. Several factors, such as inbreeding and fragmented population structure due to the lack of genetic transfer between populations, can contribute to this. The average observed heterozygosity level (0.657; Table1) was similar to those of the Indian leopard (*P.p. fusca*) (0.696) and Persian leopard (*P.p. saxilcolor*) (0.616) populations (Uphyrkina et al. 2001). Other studies have reported that the heterozygosity in Indian leopards varies between 0.57 and 0.74 (Mondol et al. 2009; Dutta et al. 2013). These differences in levels of heterozygosity may be due to different sample size and sample distribution. As a result of the continuous distribution of African leopards, high levels of heterozygosity (0.77–0.80) were observed in studies undertaken in central Africa (Spong et al. 2000; Uphyrkina et al. 2001), while the lowest average heterozygosity was recorded in the endangered Far Eastern leopard (*P.p. orientalis*) (0.356) (Uphyrkina et al. 2001).

Our sample size was low, and this may have reduced the potential for identifying higher rates of gene flow between populations and made it difficult to determine recolonization of historically extirpated populations. However, leopard population density has been estimated at between 680 and 900 individuals (J. S. McManus et al. unpubl. data) based on predicted leopard habitat for the Eastern and Western Cape (Swanepoel et al. 2013; J. S. McManus et al. unpubl. data). Our sample represents 4–6% of the leopard population, which is more than previous DNA studies in Africa (0.18%) (Spong et al. 2000). Hale et al. (2012) found that, for microsatellites, sample sizes of above 25 to 30 showed minimal variability in allele frequency and expected heterozygosity, suggesting our sample size was suitable to infer genetic structure and gene flow.

### Conservation management implications

Population subdivision may lead to decreased genetic variation within individual subpopulations owing to genetic drift (Lande and Barrowclough 1987); thus, the three subpopulations studied require genetic transfer to remain as one evolutionary unit. Our estimates of gene flow, presented as relative measures of connectivity between populations, provide a useful index to assist management. The north population had low immigration and emigration (0.57–0.51 migrants/generation), with higher gene flow recorded between the central and south subpopulations (3.68 migrants/generation). The levels of gene flow are low compared with results for other carnivores (Cegelski et al. 2003; Dutta et al. 2013), which highlights the need for further research and active conservation management. The low gene flow estimates furthermore have important implications for human-caused mortality, particularly where human–carnivore conflict exists and harvesting is practiced.

It has been proposed that only one migrant per generation is needed to prevent population differentiation (Kimura and Ohta 1971; Vucetich and Waite 2000); however, recent evidence suggests 10 or more migrants per generation is more realistic for natural populations (Mills and Allendorf 1996). Population viability model predictions for other large solitary felids such as cougars (*Puma concolor*) in a human-dominated landscape indicated that, even when high immigration rates were used in models, small populations became extinct within 100 years (Sweanor et al. 2000). Additionally, to withstand the threat of extinction over more than 100 years, continuous habitat had to be between 1000 and 2200 km². Considering these parameters, all three of the observed leopard subpopulations are at risk of extinction.

To ensure gene flow between populations, habitat connectivity and opportunities for genetic movement between discontinuous populations are essential (Ernest et al. 2003; Dutta et al. 2013). Finding solutions to human–carnivore conflict (see McManus et al. 2014) may reduce carnivore mortality and increase genetic transfer between populations. Active harvesting of leopards in the observed substructured populations with moderate to low gene flow will increase the risk of extinction.

The detection of population divergence in leopard populations in South Africa indicates an increasingly fragmented landscape for carnivores. As the human population continues to increase rapidly, the need to maintain connectivity of natural populations is becoming greater. The conservation implications with this genetic index can be useful to conservation biologists. To ensure population persistence of carnivores, their management requires identifying and securing leopard habitat, promoting habitat connectivity, considering local translocations as opposed to killing individuals, curbing human-carnivore conflict and ensuring future development considers species-specific alternatives to ensure connectivity.

## References

[b1] Anderson CD, Epperson BK, Fortin MJ, Holderegger R, James P, Rosenberg MS (2010). Considering spatial and temporal scale in landscape-genetic studies of gene flow. Mol. Ecol.

[b3] Bailey TN (1993). The African leopard: ecology and behaviour of a solitary felid.

[b4] Balkenhol N, Waits LP, Dezzani RJ (2009). Statistical approaches in landscape genetics: an evaluation of methods for linking landscape and genetic data. Ecography.

[b5] Beier P, McCullough DR (1995). Metapopulation models, tenacious tracking and cougar conservation. Metapopulations and wildlife conservation.

[b6] Blair C, Weigel DE, Balazik M, Keeley AT, Walker FM, Landguth E (2012). A simulation-based evaluation of methods for inferring linear barriers to gene flow. Mol. Ecol. Resour.

[b7] Carroll C, Noss RF, Paquet PC (2001). Carnivores as focal species for conservation planning in the Rocky Mountain region. Ecol. Appl.

[b8] Cegelski CC, Waits LP, Anderson NJ (2003). Assessing population structure and gene flow in Montana wolverines (*Gulo gulo*) using assignment-based approaches. Mol. Ecol.

[b9] Dutta T, Sharma S, Maldonado JE, Wood TC, Panwar HS, Seidensticker J (2013). Fine-scale population genetic structure in a wide-ranging carnivore, the leopard (Panthera pardus fusca) in central India. Divers. Distrib.

[b10] Epps CW, Palsbøll PJ, Wehausen JD, Roderick GK, Ramey R R, McCullough DR (2005). Highways block gene flow and cause a rapid decline in genetic diversity of desert bighorn sheep. Ecol. Lett.

[b11] Ernest HB, Boyce WM, Bleich VC, May B, Stiver SJ, Torres SG (2003). Genetic structure of mountain lion (*Puma concolor*) populations in California. Conserv. Genet.

[b13] Frankham R (2005). Genetics and extinction. Biol. Conserv.

[b14] Gavashelishvili A, Lukarevskiy V (2008). Modelling the habitat requirements of leopard *Panthera pardus* in west and central Asia. J. Appl. Ecol.

[b15] Ginsberg JR, Milner-Gulland EJ (1994). Sex-biased harvesting and population dynamics in ungulates: implications for conservation and sustainable use. Conserv. Biol.

[b17] Groom MJ, Meffe GK, Carroll CR (2006). Principles of conservation biology.

[b18] Guillot G, Mortier F, Estoup A (2005). GENELAND: a computer package for landscape genetics. Mol. Ecol. Notes.

[b19] Hale ML, Burg TM, Steeves TE (2012). Sampling for microsatellite-based population genetic studies: 25 to 30 individuals per population is enough to accurately estimate allele frequencies. PLoS One.

[b20] Hanski I, Gilpin M (1991). Metapopulation dynamics: brief history and conceptual domain. Biol. J. Linn. Soc.

[b21] Harrison S (1991). Local extinction in a metapopulation context: an empirical evaluation. Biol. J. Linn. Soc.

[b22] Harrison S, Gilpin AD, Taylor AD, Hanski I (1997). Empirical evidence for metapopulation dynamics: a critical review. Metapopulation biology: ecology, genetics, and evolution.

[b23] Holderegger R, Di Giulio M (2010). The genetic effects of roads: a review of empirical evidence. Basic Appl. Ecol.

[b24] Keller LF, Waller DM (2002). Inbreeding effects in wild populations. Trends Ecol. Evol.

[b25] Kimura M, Ohta T (1971). Theoretical aspects of population genetics (Vol. 4).

[b26] Lande R, Barrowclough GF (1987). Effective population size, genetic variation, and their use in population management. Viable Popul. Conserv.

[b27] Levins R (1970). Extinction. Lect. Math. Life Sci.

[b29] Loxterman JL (2011). Fine scale population genetic structure of pumas in the Intermountain West. Conserv. Genet.

[b31] Martins N (2006). Conservation genetics of *Panthera pardus*.

[b32] McManus JS, Dickman A, Gaynor D, Smuts B, MacDonald DW (2014). Dead or Alive? Comparing the costs and benefits of lethal and non-lethal human-wildlife conflict mitigation methods on commercial farms. Oryx.

[b33] McRae BH, Beier P, Dewald LE, Huynh LY, Keim P (2005). Habitat barriers limit gene flow and illuminate historical events in a wide-ranging carnivore, the American puma. Mol. Ecol.

[b34] Melnick DJ, Hoelzer GA (1992). Difference in male and female macaque dispersal lead to contrasting distributions of nuclear and mitochondrial DNA variation. Int. J. Primatol.

[b35] Menotti-Raymond MA, O'Brien SJ (1995). Evolutionary conservation of ten microsatellite loci in four species of Felidae. J. Hered.

[b36] Mills LS, Allendorf FW (1996). The one-migrant-per-generation rule in conservation and management. Conserv. Biol.

[b37] Miththapala S, Seidensticker J, O'Brien SJ (1996). Phylogeographic subspecies recognition in leopards (*Panthera pardus*): molecular genetic variation. Conserv. Biol.

[b38] Mondol S, Navya R, Athreya V, Sunagar K, Selvaraj V, Ramakrishnan U (2009). A panel of microsatellites to individually identify leopards and its application to leopard monitoring in human dominated landscapes. BioMed Central Genet.

[b39] Nei M (1978). Estimation of average heterozygosity and genetic distance from a small number of individuals. Genetics.

[b40] Noss RF, Carroll C, Vance-Borland K, Wuerthner G (2002). A multicriteria assessment of the irreplaceability and vulnerability of sites in the Greater Yellowstone Ecosystem. Conserv. Biol.

[b42] O'Brien SJ, Johnson WE (2005). Big cat genomics. Genomics Hum. Genet.

[b43] Peakall R, Smouse PE (2006). GenAlEx 6: genetic analysis in Excel. Population genetic software for teaching and research. Mol. Ecol. Notes.

[b44] Pilot M, Jedrzejewski W, Branicki W, Sidorovich VE, Jedrzejewska B, Stachura K (2006). Ecological factors influence population genetic structure of European grey wolves. Mol. Ecol.

[b45] Powell RA, Zimmerman JW, Seaman DE, Gilliam JF (1996). Demographic analyses of a hunted black bear population with access to a refuge. Conserv. Biol.

[b46] Pritchard JK, Stephens M, Donnelly P (2000). Inference of population structure using multilocus genotype data. Genetics.

[b47] Raymond M, Rousset F (1995). GENEPOP (version 1.2): population genetics software for exact tests and ecumenicism. J. Hered.

[b48] Schaffer ME (1988). Evolutionarily stable strategies for a finite population and a variable contest size. J. Theor. Biol.

[b50] Seutin G, White BN, Boag PT (1991). Preservation of avian blood and tissue samples for DNA analyses. Can. J. Zool.

[b52] Sinclair EA, Swenson EL, Wolfe ML, Choate DC, Bates B, Crandall KA (2001). Gene flow estimates in Utah's cougars imply management beyond Utah. Anim. Conserv.

[b53] Slatkin M (1987). Gene flow and the geographic structure of natural populations. Science.

[b54] Sork VL, Nason J, Campbell DR, Fernandez FJ (1999). Landscape approaches to historical and contemporary gene flow in plants. Trends Ecol. Evol.

[b55] Spong G, Johansson M, Björklund M (2000). High genetic variation in leopards indicates large and long-term stable effective population size. Mol. Ecol.

[b56] Stenseth NC, Ehrich D, Rueness EK, Lingjærde OC, Chan KS, Boutin S (2004). The effect of climatic forcing on population synchrony and genetic structuring of the Canadian lynx. Proc. Natl Acad. Sci. USA.

[b57] Sunquist ME, Sunquist F (2002). Wild cats of the world.

[b58] Swanepoel LH, Lindsey P, Somers MJ, Hoven WV, Dalerum F (2013). Extent and fragmentation of suitable leopard habitat in South Africa. Anim. Conserv.

[b59] Sweanor LL, Logan KA, Hornocker MG (2000). Cougar dispersal patterns, metapopulation dynamics, and conservation. Conserv. Biol.

[b60] Teske PR, Papadopoulos I, Barker NP, McQuaid CD (2012). Mitrochondrial DNA paradox: sex-specific genetic structure in a marine mussel – despite maternal inheritance and passive dispersal. BioMed Central Genet.

[b61] Uphyrkina O, Johnson WE, Quigley H, Miquelle D, Marker L, Bush M (2001). Phylogenetics, genome diversity and origin of modern leopard, *Panthera pardus*. Mol. Ecol.

[b62] Van Oosterhout C, Hutchinson WF, Wills DP, Shipley P (2004). Micro-checker: software for identifying and correcting genotyping errors in microsatellite data. Mol. Ecol. Notes.

[b63] Vucetich JA, Waite TA (2000). Is one migrant per generation sufficient for the genetic management of fluctuating populations?. Anim. Conserv.

[b64] Wahland S (1928). Zusammersetung von Populationen und Korrelation-sercheinungen von Standpunkt der Verebungslehre aus betrachtet. Hereditas.

[b65] Walker CW, Harveson LA, Pittman MT, Tewes ME, Honeycutt RL (2000). Microsatellite variation in two populations of mountain lions (*Puma concolor*) in Texas. Southwest. Nat.

[b66] Wayne RK, L Gittleman J, Koepfli KP (1996). Demographic and historical effects on genetic variation of carnivores. Carnivore behavior, ecology, and evolution.

[b67] Weir BS, Cockerham CC (1984). Estimating F-statistics for the analysis of population structure. Evolution.

[b68] Wielgus RB, Bunnell FL (1994). Dynamics of a small, hunted brown bear (Ursus arctos) population in Southwestern Alberta, Canada. Biol. Conserv.

[b69] Woodroffe R, Ginsberg JR (1998). Edge effects and the extinction of populations inside protected areas. Science.

[b70] Woodroffe R, Thirgood S, Woodroffe R, Thirgood S, Rabinowitz A, Rabinowitz A (2005). People and wildlife: conflict or coexistence?. The impact of human-wildlife conflict on natural systems.

[b71] Wright S (1984). Evolution and the genetics of populations. Experimental results and evolutionary deductions.

[b72] Zhang D, Hewitt GM (2003). Nuclear DNA analyses in genetic studies of populations: practice, problems and prospects. Mol. Ecol.

